# Early diagnosis and management of cardiac manifestations in mucopolysaccharidoses: a practical guide for paediatric and adult cardiologists

**DOI:** 10.1186/s13052-018-0560-3

**Published:** 2018-11-16

**Authors:** Lucia Boffi, Pierluigi Russo, Giuseppe Limongelli

**Affiliations:** 1 U.O. Cardiologia ASST Monza, 20900 Monza, Italy; 2 Center of Paediatric Cardiology and Adult Cardiac Genetic Disease, ASL BT, Via Padre Pio, 76125 Trani, (BT) Italy; 3 U.O. Cardiologia Pediatrica e U.O. di Riabilitazione e Scompenso Cardiaco, Ospedale Monaldi, A.O. dei Colli, Dipartimento di Scienze Cardio-Toraciche e Respiratorie, Università della Campania Luigi Vanvitelli, Via Leonardo Bianchi, 80131 Naples, Italy; 4 Institute of Cardiovascular Sciences, University College of London, Paul O’Gorman Building, 72 Huntley Street, London, WC1E 6DD UK; 5 Department of Cardiothoracic Sciences, Università della Campania Luigi Vanvitelli, Via Leonardo Bianchi, 80131 Naples, Italy

**Keywords:** Mucopolysaccharidoses (MPS), Cardiac disease, Valve disease, Coronary artery disease, Aortopathy

## Abstract

Mucopolysaccharidoses (MPS) are a group of hereditary disorders caused by lysosomal storage of glycosaminoglycans (GAGs) and characterized by a wide variability of phenotypes from severe fetal-neonatal forms to attenuated diseases diagnosed in adult individuals. The clinical picture generally worsens with age due to progressive storage involving mucosal tissue, upper airways and lungs, bones and joints, central and peripheral nervous system, heart, liver, eye, and ear. Cardiac storage of GAGs involves valves, heart muscle, and vessels (particularly the coronary arteries), and can be specific in relation to different MPS types and enzyme defects. MPS I, II, and VI are those with the most severe cardiac involvement. The cardiologist is a key figure in MPS, and their role is expanding from cardiac-specific management to early diagnosis when the mild disease phenotypes have not yet been recognized by other specialists. Familial and personal history, electrocardiography, imaging, and laboratory findings represent important steps in the clinical investigation of these patients. New treatments have led to an increased need for cardiologists to be on the lookout for MPS patients since they can significantly improve the lives of people with MPS if they suspect the diagnosis and refer them for enzyme replacement therapy or bone marrow transplantation.

## Background

Mucopolysaccharidoses (MPS) are a group of hereditary disorders caused by lysosomal storage of glycosaminoglycans (GAGs) [1, 2]. They are characterized by a wide variability of phenotype, from severe fetal-neonatal forms to attenuated diseases diagnosed in adult individuals. The phenotype generally worsens with age due to progressive storage involving mucosal tissue, upper airways and lungs, bones and joints, central and peripheral nervous system, heart, liver, eye, and ear. Cardiac storage of GAG involves valves, heart muscle, and vessels (particularly the coronary arteries), and can be specific in relation to different MPS types and enzyme defects [1, 2]. MPS I, II, and VI are those with the most severe cardiac involvement [1, 2]. Recently new treatments, such as haematopoietic stem cell transplantation (HSCT) and enzyme replacement therapy (ERT), have become available for many of these disorders and thus there is now an urgency for early diagnosis to allow access to such therapies [1, 2]. Thanks to the new therapies, MPS patients live longer than in the past and the need for organ-specific management and palliative treatment is progressively increasing. Although a specific effect on severe valve defects is not clearly demonstrated, ERT has been demonstrated to reduce left ventricular mass in patients with cardiomyopathy [1, 2]. Also, HSCT has a systemic effect and the ability to reduce the progression of cognitive delay [1, 2]. A combination of pre-transplant ERT and HSCT has been suggested in MPS I patients with severe cardiomyopathy [3].

The cardiologist is a key figure in MPS, and their role is expanding from cardiac-specific management to early diagnosis when the mild disease phenotypes have not been recognized yet by other specialists. The mainstay of MPS, as for all lysosomal storage diseases, is “early diagnosis-early management”, and a correct interpretation of clinical and laboratory findings and of typical electrocardiography (ECG) and imaging features in these patients may help in making the clinical diagnosis.

### Diagnosis

**Fig. 1:**
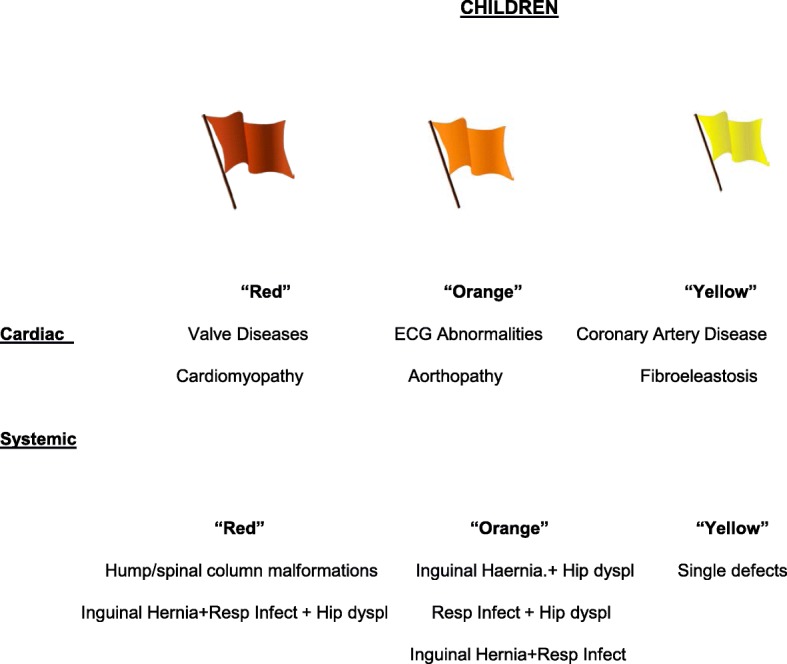
Cardiac and systemic hallmarks/flags (red, orange, yellow) for the clinical diagnosis of MPS in children. Definite diagnosis: combination of cardiac + systemic red flags; probable diagnosis: combination of cardiac + systemic orange flags; possible diagnosis: combination of cardiac + systemic yellow flags. This classification is based on expert points of view and prevalence of defects. To date, no data are available on sensitivity and specificity of single and composed clinical hallmarks of the disease. ECG electrocardiography, dyspl dysplasia, Resp Infect respiratory infections

**Fig. 2:**
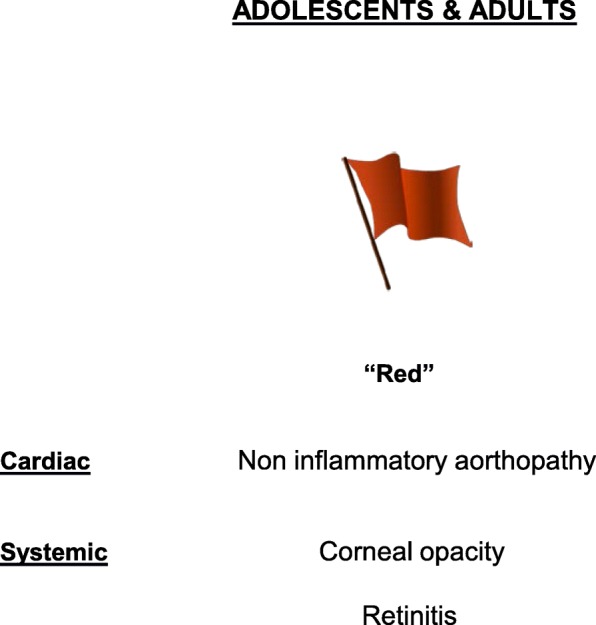
Cardiac and systemic hallmarks/flags (red) for the clinical diagnosis of MPS in adolescents/adults

The diagnosis of MPS is based on cardiac and non-cardiac disease manifestations. Red flags for the diagnosis has been proposed (Table 1, Figs. 1 and 2) [2].

**Table 1:** Ten-point checklist for cardiologists to suspect mucopolysaccharidosis

1. Family history: consanguinity and/or X-linked inheritance (female to male; no male to male transmission)	Yes/No
2. Hump/spinal column malformations	Yes/No
3. Hip dysplasia	Yes/No
4. Inguinal hernia	Yes/No
5. Respiratory infections	Yes/No
6. Facial dysmorphisms	Yes/No
7. Corneal opacity/retinitis	Yes/No
8. Valve disease (mitral/aortic)	Yes/No
9. Electrocardiography: atrioventricular block	Yes/No
10. Aortopathy/coronary artery disease	Yes/No

#### Is a family history/pedigree relevant for the cardiologist?

All MPS, except type II which is X-linked, are autosomal recessive disorders. Thus, family pedigree can be apparently silent while other potential “red flags” should be considered, including consanguinity and ethnic background (small inbreeding communities such as religious groups (i.e. Hutterites, Amish) or isolated populations in mountain villages). Male-to-male transmission excludes a diagnosis of MPS.

In summary, ethnic background and pedigree (i.e. consanguinity) should be considered in the diagnostic flow-chart of MPS.

#### Is clinical examination relevant for the cardiologist?

Clinical examination can be relevant to raise a suspicion of MPS. Evidence of a cardiac murmur (aortic, mitral) in the presence of typical dysmorphic features (coarse face), skeletal, and joint abnormalities is highly suggestive of MPS that should be differentiated from other genetic disorders (i.e. RAS-MAPK disease, including Noonan, LEOPARD, Costello, and Cardiofaciocutaneous syndrome). It is important to underline that the absence of a cardiac murmur and/or one or more systemic features does not exclude the diagnosis per se due to the extreme heterogeneity of the phenotype (especially in adult patients with milder forms) [4].

In summary, clinical examination is useful to exclude a syndromic and/or metabolic form of valve disease and/or cardiomyopathy.

#### Is ECG useful in patients with MPS?

**Fig. 3:**
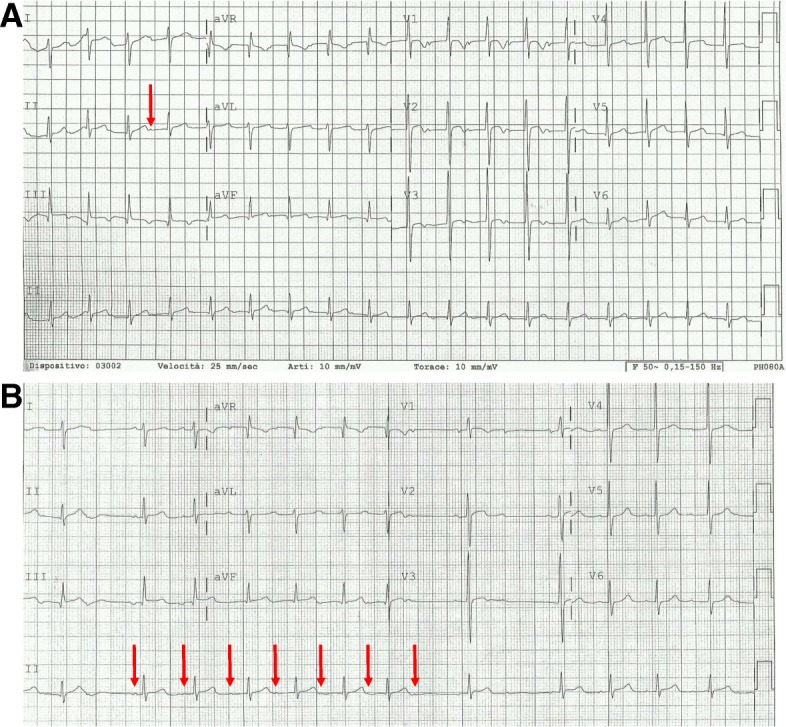
MPS I in **a** 14-year-old male. a First-degree atrioventricular block: PR interval greater than 0.20 s. **b** Second-degree atrioventricular block type 1: progressive prolongation with dropped beat. The red arrows indicate the P wave

Conduction abnormalities have been reported with variable rates in patients with different MPS subtypes [1, 3, 5]. Atrioventricular blocks (AVBs) can be progressive, and patients requiring an aggressive approach with permanent pacing and autoptic findings of GAG infiltration in sudden death cases have been reported in the literature (Fig. 3) [6–8].

In summary, ECG is mandatory in patients with MPS. AVBs can be common in some specific MPS subtypes, but they are rarely progressive.

#### Is imaging useful in MPS?

**Fig. 4:**
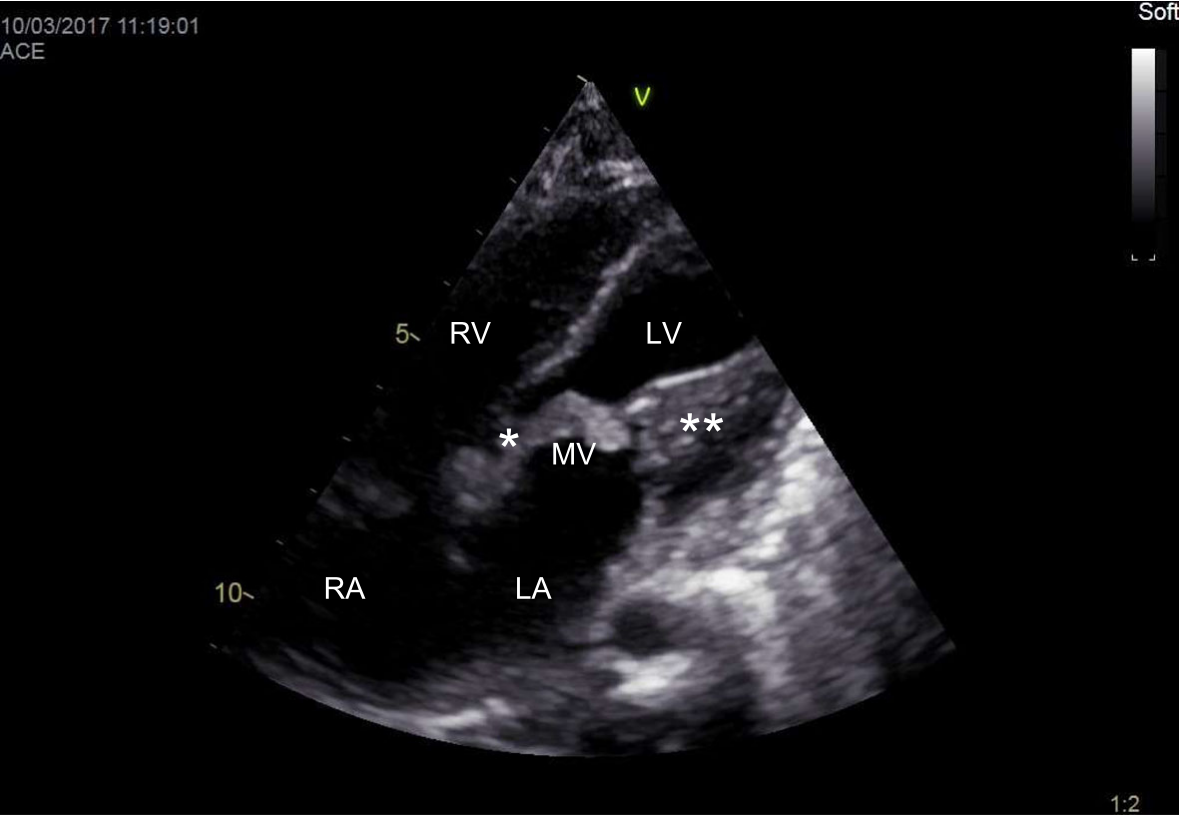
Mitral valve stenosis in an MPS VI 16-year-old male. *Anterior mitral leaflet; **mitral papillary muscle. LA left atrium, LV left ventricle, MV mitral valve, RA right atrium, RV right ventricle

**Fig. 5:**
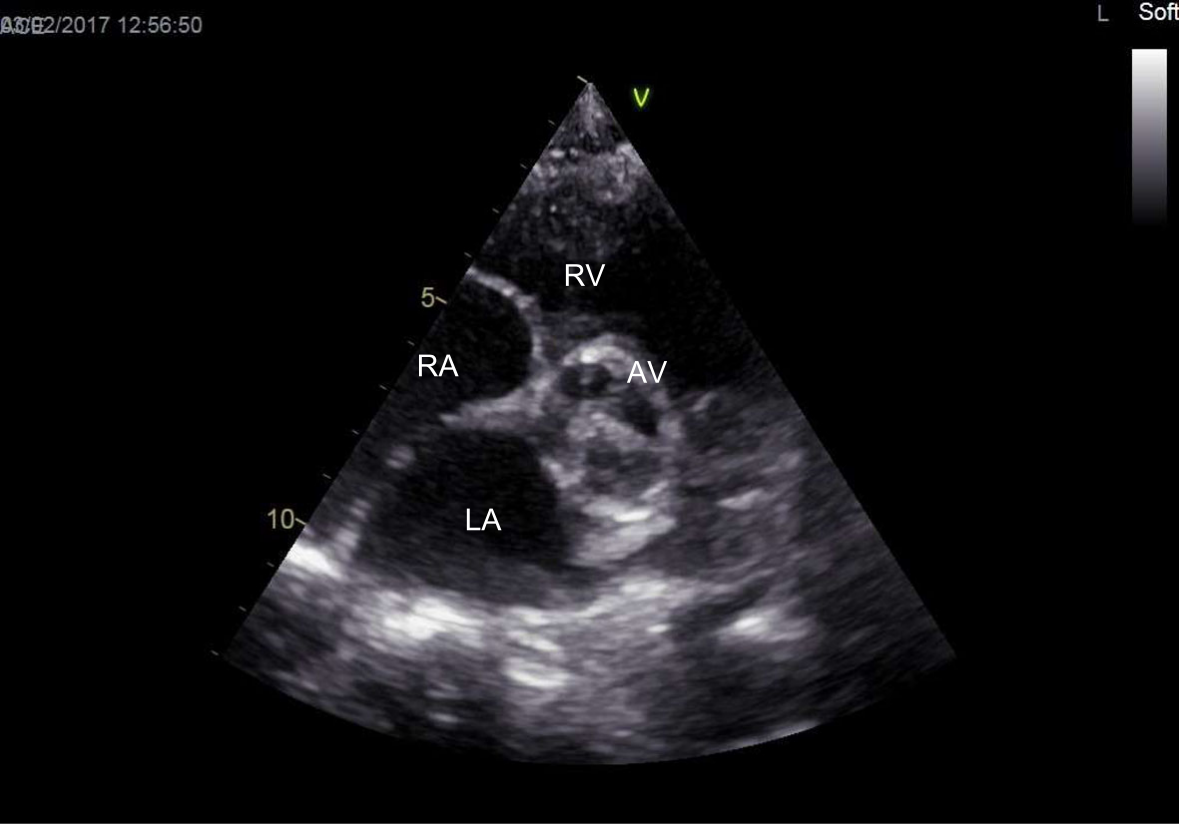
Aortic valve stenosis in an MPS II 20-year-old male. AV aortic valve, LA left atrium, RA right atrium, RV right ventricle

**Fig. 6:**
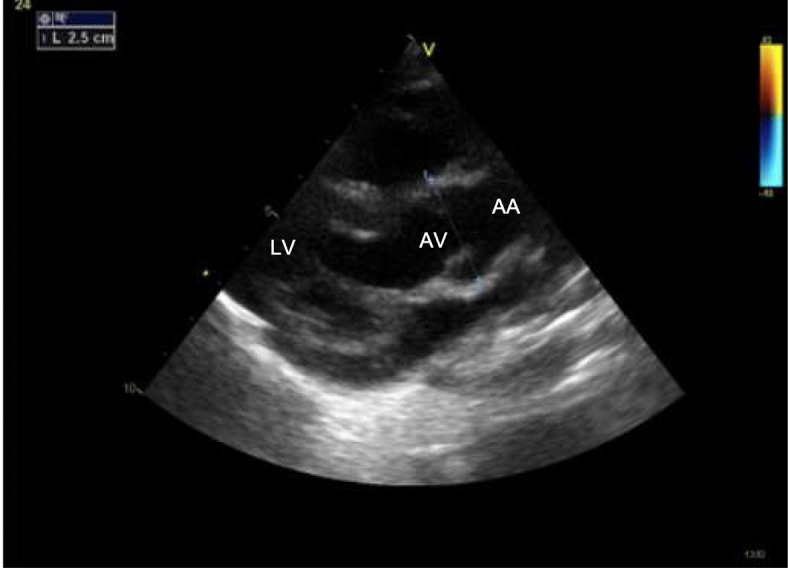
Aortic root dilation in an MPS I 5-year-old male. AA ascending aorta, AV aortic valve, LV left ventricle

M-mode two-dimensional Doppler echocardiography is the gold standard for the diagnosis of cardiac involvement in MPS, and new technologies (speckle tracking) may even improve detection of subclinical functional abnormalities [9, 10]. Valve damage in MPS is quite common and typical [1, 2, 11, 12]. At the early stage of the disease (GAG storage) it is possible to observe redundant and slightly thickened leaflets [11, 12]. The functional consequence is a regurgitation that worsens with progression of the disease when the leaflets became more thickened and fibrotic (Fig. 4) [11, 12]. End-stage disease is characterized by leaflets and subvalvular apparatus calcification and stiffness, causing valve stenosis (Fig. 5) [11, 12]. Intramyocardial infiltration of GAGs (pseudohypertrophy) may be present, although heart muscle thickening in MPS is less important than in other storage diseases, such as Pompe disease or Anderson-Fabry disease. Thus, it is important to regularly measure wall thickness, left ventricular dimensions, and cardiac mass. All values must be normalized for body surface or, better, for body surface and age (*z* score) (www.parameterz.com; http://parameterz.blogspot.it/). Endocardial fibroelastosis is a rare and serious presentation of infantile MPS [1, 2]. Typically, the endocardium appears fibrotic with a dense echogenic appearance. Dilatation and reduced elasticity of the ascending aorta have been described (Fig. 6) [1, 2]. This is related to the harmful effect of GAGs on the formation of tropoelastin. Congenital defects, such as aortic coarctation, has been rarely reported [1, 2]. Axial computed tomography is rarely used in MPS patients (a large radiation exposure) but may be considered in selected cases for the study of coronary artery disease. Furthermore, magnetic resonance imaging (MRI) is rarely used in MPS but it may be considered when echo windows are inadequate to evaluate cardiac function and structure in patients with moderate to severe disease. Coronary angiography may be indicated when a strong suspicion of coronary artery disease is present, although the sensitivity may be low and may miss significant disease due to the diffuse nature of the coronary artery process in MPS [1–3].

In summary, cardiac imaging is essential for diagnosis and follow-up of MPS patients. Echocardiography is the most important imaging tool and the gold standard in these patients.

#### Are laboratory measures useful in MPS?

Brain natriuretic peptide (BNP) and its precursor NT-proBNP may be helpful for distinguishing between cardiac and pulmonary dyspnoea, which is often a dilemma in MPS patients (from personal experience). BNP may also be helpful to establish when to start and/or how to monitor cardiac therapy in patients with moderate to severe mitral or aortic regurgitation.

In summary, BNP and NT-proBNP can be considered in MPS patients to distinguish between cardiac and pulmonary disease (since they often coexist), and to guide cardiac therapy.

### Management

#### How to manage valve disease in MPS?

In patients with MPS, valve involvement is remarkable as it is present in almost all patients with MPS I and II and, to a lesser extent, in patients with other forms of MPS. Moreover, valvular disease usually has an early onset, sometimes rapid progression, and a high degree of anatomical complexity [1, 2, 9, 11–13]. In fact, both the valvular and the subvalvular apparatus display anomalies caused by the accumulation of GAGs.

Therefore, patients with MPS require a cardiac examination at the time of diagnosis, even at a paediatric age. Cardiac evaluation should always include an echocardiography to define the involvement of the valvular apparatus and its extent. Cardiological follow-up should be performed yearly for mild or stable valvular disease, and at least twice a year for patients with severe valvular disease with signs of suboptimal heart function. These patients should be treated with heart failure drugs according to conventional guidelines. Follow-up should be even more frequent in cases with signs of heart failure to optimize drug therapy or to define a surgical indication.

As reported in the literature, the management of the majority of MPS patients with valvular disease has been, up to now, mostly conservative. The reasons for conservative strategies are various, including the overall perioperative risk of surgery in early, severe MPS forms, but also the fact that most patients limit their activity (and therefore may have minor symptoms related to their valve disease), or the valve disease is mild or not severe enough to require surgery (as often happen in adult patients) [14, 15].

A surgical intervention for valvular replacement is therefore indicated in: a) symptomatic patients without significant comorbidities, and with a chest shape not limiting intubation (pectus excavatum and pectus carinatum); and b) asymptomatic patients with severe valvular diseases and with evident sign of disease progression (systolic function impairment, chamber dilation, pulmonary hypertension, arrhythmias).

Surgery should be performed in specialized centres that are used to treating this kind of patient. Until now, patients who underwent cardiac surgery received mechanical protheses; however, considering the longer life expectancy of these patients and the diffusion of percutaneous valve technology, new scenarios could be opened in the future [16]. At the moment, however, the literature on this topic is scarce.

In summary, patients with mild or stable valvular disease should receive clinical evaluation, ECG, and echocardiography once a year. Patients with severe valvular disease should receive clinical evaluation, ECG, and echocardiography twice a year. Patients with symptoms of heart failure not controlled by pharmacological therapy should receive clinical evaluation, ECG, and echocardiography every 3–6 months. Valvular disease should be treated with a conservative approach. When surgery is indicated (symptomatic patients or asymptomatic patients with severe, progressive disease), a multidisciplinary approach (“heart team” evaluation) is required in experienced centres.

#### How to manage cardiomyopathy in MPS?

Early onset of a dilatative cardiomyopathy in patients with MPS is considered a hallmark of the aggressive form of the disease and of severe cardiac impairment [3]. Hospitalization is usually required to optimize pharmacological therapy and/or to support circulation in a life-threatening presentation (intravenous inotropic support). Progressive, hypokinetic cardiomyopathy should raise the suspicion of an underlying ischemic disease. Prognosis is usually ominous.

When the cardiomyopathy is secondary to valvular disease, the clinical course is different and less severe. In these patients, pharmacological therapies can prevent or reduce the risk of heart failure, and this presentation is considered an indication for surgery.

In summary, cardiomyopathy without valvular disease is a rare presentation of very severe forms of MPS. Cardiomyopathy is more frequently associated with valvular disease, with a later onset, often requiring pharmacological treatment, or in some cases valve replacement.

#### How to manage coronary artery disease in MPS?

Coronary artery disease is frequently reported in the autopsies of patients with MPS [17]. However, in the clinical practice, symptoms of chest pain are almost absent, and the real prevalence of the disease is unknown. When coronary heart disease is suspected, a stress test is hardly feasible; therefore, more information could be obtained by other tests (i.e. ECG Holter, stress echo, or MRI, when feasible).

In patients with a planned valve replacement, coronary angiography is mandatory. Coronary angiography (with or without intravascular ultrasound) should be considered when non-invasive tests suggest the presence of ischaemic disease (repolarization abnormalities, ventricular arrhythmias, progressive systolic impairment).

In summary, coronary artery disease is often clinically “silent” in patients with MPS. In patients with a planned valve replacement, coronary angiography is mandatory before valve replacement, and it should be considered when a strong suspicion of ischaemic heart disease (repolarization abnormalities, ventricular arrhythmias, progressive systolic impairment) has been raised.

#### How to manage aortopathy in MPS?

Data on vascular complications and aortopathy in MPS and other storage disorders are only just appearing in the literature. No specific indications for surgery have been suggested for MPS patients, and surgery should be discussed case-by-case according to current guidelines, the presence of coexisting cardiac lesions, and the severity of the systemic phenotype. An interesting perspective comes from mouse model studies, i.e. the potential use of losartan, an angiotensin-1 receptor antagonist, to treat aortopathy in patients with MPS (as in patients with aortopathy related to Marfan syndrome) [18].

In summary, aortopathy is an emerging problem in MPS patients. Angiotensin-1 receptor blockers represent a promising therapy based on experimental studies.

## Conclusions

Cardiac involvement is part of the clinical spectrum in patients with MPS, mostly involving types I, II, and VI. Valve disease is the most common cardiac defect at disease onset, while dilated cardiomyopathy and coronary involvement are rarer. Cardiologists should be aware of the clinical features and natural history of the disease, since early diagnosis and management are important in the most severe presentations.

## Abbreviations

AVB: Atrioventricular block
BNP: brain natriuretic peptide
ECG: Electrocardiography
ERT: Enzyme replacement therapy
GAG: glycosaminoglycan
HSCT: Haematopoietic stem cell transplantation
MPS: Mucopolysaccharidosi(e)s
MRI: Magnetic resonance imaging
NT-proBNP: N-terminal pro brain natriuretic peptide
